# Supplementary material for: Causal Relationship between Aging and Anorexia Nervosa: A White-Matter-Microstructure-Mediated Mendelian Randomization Analysis
Source: Biomedicines. 2024 Aug 16;12(8):1874. doi: 10.3390/biomedicines12081874 (PMC11351342; doi:10.3390/biomedicines12081874)
Supplement: Supplementary file 1 [file biomedicines-12-01874-s001.zip › Supplementary Figure/Figure S4/Singe SNP plot of MR analysis for TL to ubm-b-1595.pdf]

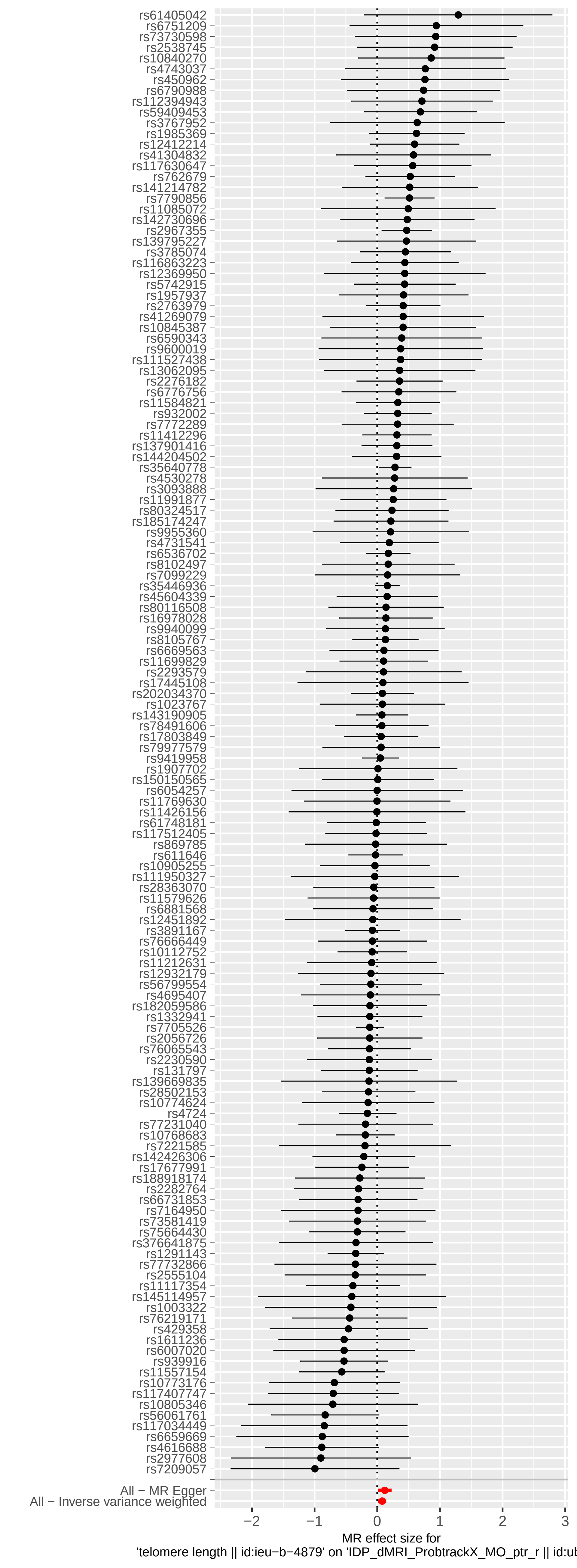
rs61405042
rs6751209
rs73730598
rs2538745
rs10840270
rs4743037
rs450962
rs6790988
rs112394943
rs59409453
rs3767952
rs1985369
rs12412214
rs41304832
rs117630647
rs762679
rs141214782
rs7790856
rs11085072
rs142730696
rs2967355
rs139795227
rs3785074
rs116863223
rs12369950
rs5742915
rs1957937
rs2763979
rs41269079
rs10845387
rs6590343
rs9600019
rs111527438
rs13062095
rs2276182
rs6776756
rs11584821
rs932002
rs7772289
rs11412296
rs137901416
rs144204502
rs35640778
rs4530278
rs3093888
rs11991877
rs80324517
rs185174247
rs9955360
rs4731541
rs6536702
rs8102497
rs7099229
rs35446936
rs45604339
rs80116508
rs16978028
rs9940099
rs8105767
rs6669563
rs11699829
rs2293579
rs17445108
rs202034370
rs1023767
rs143190905
rs78491606
rs17803849
rs79977579
rs9419958
rs1907702
rs150150565
rs6054257
rs11769630
rs11426156
rs61748181
rs117512405
rs869785
rs611646
rs10905255
rs111950327
rs28363070
rs11579626
rs6881568
rs12451892
rs3891167
rs76666449
rs10112752
rs11212631
rs12932179
rs56799554
rs4695407
rs182059586
rs1332941
rs7705526
rs2056726
rs76065543
rs2230590
rs131797
rs139669835
rs28502153
rs10774624
rs4724
rs77231040
rs10768683
rs7221585
rs142426306
rs17677991
rs188918174
rs2282764
rs66731853
rs7164950
rs73581419
rs75664430
rs376641875
rs1291143
rs77732866
rs2555104
rs11117354
rs145114957
rs1003322
rs76219171
rs429358
rs1611236
rs6007020
rs939916
rs11557154
rs10773176
rs117407747
rs10805346
rs56061761
rs117034449
rs6659669
rs4616688
rs2977608
rs7209057
All − MR Egger
All − Inverse variance weighted
−2
−1
0
1
2
3
MR effect size for
'telomere length || id:ieu−b−4879' on 'IDP_dMRI_ProbtrackX_MO_ptr_r || id:ub
